# The Formation of 3-Monochloropropanediol Esters and Glycidyl Esters during Heat-Induced Processing Using an Olive-Based Edible Oil

**DOI:** 10.3390/foods11244073

**Published:** 2022-12-16

**Authors:** Yingrui Ji, Dongming Lan, Weifei Wang, Kok Ming Goh, Chin Ping Tan, Yonghua Wang

**Affiliations:** 1 School of Food Science and Engineering, South China University of Technology, Guangzhou 510640, China; 2 Sericultural and Agri-Food Research Institute, Guangdong Academy of Agricultural Sciences, Guangzhou 510610, China; 3 Department of Food Technology, Faculty of Food Science and Technology, Universiti Putra Malaysia, Seri Kembangan 43300, Malaysia; 4 Guangdong Yue-Shan Special Nutrition Technology Co., Ltd., Foshan 528000, China

**Keywords:** 3-MCPD ester, glycidyl ester, diacylglycerol, crackers, frying oil

## Abstract

With the prevalence of edible diacylglycerol (DAG) oil, which is beneficial to human, the generation of 3-monochloropropanediol esters (3-MCPDE) and glycidyl esters (GE) as well as the stability of physical properties during heat-induced processing still need to be explored. In this study, the experiment used olive-based edible oil with different contents of DAG (40, 60, and 80%) to make crackers and fry chicken. They were heated at 160 and 180 °C to determine the changes in 3-MCPDE and GE, the crackers’ hardness and gumminess, and the physical properties of the oil. During baking and frying, 3-MCPDE decreased, while the content of GE slightly increased with the prolonged heating duration. Finally, 3-MCPDE and GE were lower than 1.25 mg/kg and 1.00 mg/kg, respectively. The AV increased proportionally as duration increased and POV was below 0.30 g/100 g. In general, the changes in 3-MCPDE and GE were related to the heating temperature and duration, and not significantly (*p* > 0.05) related to the content of DAG.

## 1. Introduction

3-monochloropropanediol ester (3-MCPDE) and glycidyl esters (GE) are heat-induced processed contaminants found in refined vegetable oil [1]. GE can be metabolized into free glycidol, which is classified as “probably carcinogenic to human” (Group 2A) by the International Agency for Research on Cancer (IARC) [2]. In February 2018, the European Commission (EC) set the maximum GE limit at 1.00 mg/kg in edible oils. A stringent GE limit was 0.50 mg/kg for oils used to formulate food. Meanwhile, 3-MCPD is assessed as a non-genotoxic carcinogen at a tolerable daily intake (TDI) of 2.00 µg/kg body weight in human [3]. According to Regulation (EU) 2020/1322, the EC proposed two maximum levels for 3-MCPDE in refined oils, which were 1.25 mg/kg and 2.50 mg/kg, in September 2020.

The natural and unrefined oils do not contain 3-MCPDE or GE, and they are formed due to the high temperature during the refining deodorization step of vegetable oil [1]. Therefore, applications of edible oils, such as baking and frying, can also result in the production of 3-MCPDE and GE because of high temperature [4,5]. In addition, the content of NaCl [6], type of frying media [7], frying methods [8], frying temperature [9], antioxidants [10] and additives [11] can affect the formation of 3-MCPDE and GE. The monoacylglycerol (MAG), diacylglycerol (DAG) and triacylglycerol (TAG) also contribute to the formation of 3-MCPDE and GE [12]. Additionally, 3-MCPDE was found in savoury foods, such as salty crackers and biscuits [13,14].

Natural edible oils mainly contain TAG, and the DAG is generally less than 10% [15]. Several studies have proved that DAG has potentially beneficial properties, such as reducing body fat, having hypolipidemic activity and regulating blood sugar level [16,17]. Therefore, DAG edible oils are considered to be functional lipids. In September 2009, it was found that the GE content was above the limit of the European Union (EU) in Econa Healthy Cooking Oil (a DAG edible oil product). With a high content of DAG (6–10%), palm oil contained the highest GE among vegetable oils [15].

Generally, olive oils with high oleic acids demonstrate good frying performance because they are stable at high temperature [18]. However, the stability of an olive-based DAG was not reported in the literature. In this study, the stability of olive-based DAG oil was investigated in both baking and frying processes. The effects of DAG content, heating temperature and duration on the formation of 3-MCPDE, GE and oil qualities were evaluated.

## 2. Materials and Methods

### 2.1. Materials and Chemicals

The olive-based TAG oil was obtained from Wilmar Oleo Co., Ltd. (Dongguan, China), and the olive-based DAG oil was obtained using laboratory methods [19]. Olive-based DAG oil was prepared at 40, 60 and 80% by mixing olive-based DAG oil and olive-based TAG oil at ratios of 4:6, 6:4 and 8:2. Materials for baking and chicken for frying were obtained from a local market.

The 3-MCPDE and GE standards (1,2-dipalmitoyl-3-chloropropanediol and glycidyl palmitate) and the internal standards (1,2-bis-palmitoyl-3-chloropropanediol-d5 and glycidyl palmitate-d5) were supplied by Toronto Research Chemicals, Inc. (Toronto, ON, Canada). All of the solvents, including n-heptane, acetone, toluene, phenylboronic acid (PBA), anhydrous tetrahydrofuran (THF), sodium bromide, sulfuric acid, sodium sulfate, sodium hydrogen carbonate and methyl alcohol, were purchased from Aladdin (Shanghai, China). The high-performance liquid chromatography (HPLC)-grade reagents chloroform, methanol, acetonitrile and isopropanol were purchased from Kermel Chemical Reagent (Tianjin, China). All other reagents and solvents were analytical grade.

Before starting experiments, the acylglycerol profile of olive-based DAG oil (40, 60 and 80%) was confirmed by HPLC (Waters Corporation, Milford, CT, USA) equipped with a refractive index detector (Waters 2414, Waters Corporation, USA) and a Phenomenex Luna column (250 mm × 4.6 mm × 5 μm film thickness, Phenomenex Corporation, Torrance, ON, Canada).

### 2.2. Preparation of Crackers

The crackers were prepared with olive-based DAG oil (40, 60 and 80%) according to the American Association of Cereal Chemists Method 10-54 with minor modifications [20]. Flour (100 g), yeast (1 g), baking soda (0.5 g), skim milk (60 g), oil (20 g) and salt (1 g) were combined to form a smooth dough. Then the dough was pressed to 3 mm thickness and cut into 40 mm × 40 mm pieces. Finally, the crackers were baked at 160 and 180 °C for 12 min. In addition, the oil without materials (as control) was heated in the oven under the same conditions. The oil in the crackers was extracted with n-hexane (ultrasonic bath) to analyze the acid value (AV), peroxide value (POV), fatty acid composition, 3-MCPDE and GE.

### 2.3. Intermittent Frying Condition

A 1.5 L volume of olive-based DAG oil (40, 60 and 80% DAG) was added to the fryer and heated at 160 and 180 °C for 10 min. One frying cycle consisted of 3 min of frying and 27 min of heating without chicken. There was a total of six cycles (180 min) of frying per day continuous for 3 days without refuelling. Meanwhile, another six systems combining DAG content (40, 60 and 80%) and heating temperature (160 and 180 °C) were established without chicken as controls. Samples of 50 mL of oil were collected after frying and stored at 4 °C every day.

### 2.4. Physico-Chemical Characteristics

The hardness and gumminess of crackers were tested using a texture analyzer (TA-XT plus, Stable Micro System, Godalming, UK) equipped with a P/36 R probe. Each sample was compressed twice to 60% of its original height at 1 mm/s [21]. Each group had 10 parallel samples.

The AV (an important parameter indicating the content of free fatty acids) and POV (an indicator of the degree of oxidation of oils) were examined according to AOCS Official Method Ca 5a-40 [22] and AOCS Official Method Ja 8-87 [23], respectively.

The fatty acid composition was determined with a gas chromatography-flame ionization detector (GC-FID) (Agilent Technologies, Mississauga, ON, Canada) with CP-Sil 88 (60 m × 0.25 mm × 0.2 mm, Dikma Technologies, Beijing, China). The oil was pretreated with KOH-methanol, and then detected with GC-FID. The percentage of fatty acid was calculated using the area normalization method [19].

### 2.5. Determination of 3-MCPDE and GE

According to AOCS Official Method Cd 29a-13 [24], 100–110 mg of oil was accurately weighed in a screw cap tube. Acidified sodium bromide solution was used to convert GE into bromopropanol ester. After that, the solution was reacted with a H_2_SO_4_-methanol solution at 40 °C for 16 h, and then was derivatized using PBA. It was analyzed by gas chromatography-mass spectrometry (SIM) (TQ8050, Shimadzu, Japan) with a capillary column Equity-1 (30 m × 0.25 mm × 1 μm, Supelco, Shanghai, China).

### 2.6. Statistical Analyses

Samples were analyzed in triplicate, and standard deviation (±SD) was reported. One-way analysis of variance and the Pearson correlation test were performed using the SPSS software (version 14.0 demo, SPSS Inc., Chicago, IL, USA). The partial least squares (PLS) analysis was performed using Minitab 16 software (Minitab Inc., State College, PA, USA).

## 3. Results

### 3.1. Physico-Chemical Properties of Baking Trial

**Figure 1:**
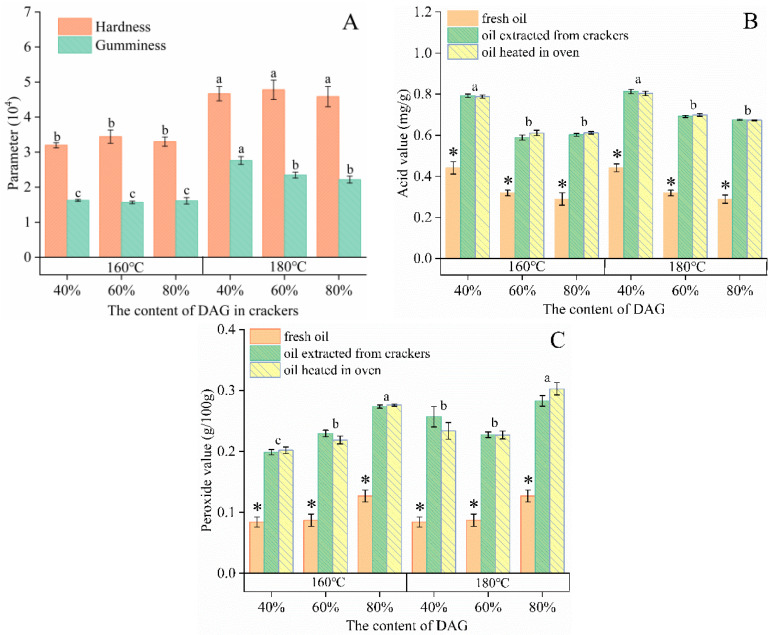
The texture (**A**) of crackers made with 40, 60 and 80% olive-based DAG oil; the AV (**B**) and POV (**C**) of oil at 160 and 180 °C. Different lowercase letters denote that values were significantly different (*p* < 0.05), and there was no significant difference (*p* > 0.05) between oil extracted from crackers and oil heated in oven. * Indicates significant difference (*p* < 0.05) between fresh oil and oil extracted from crackers or oil heated in the oven.

The hardness and gumminess of three groups of crackers (40, 60 and 80% olive-based DAG oil) were analyzed (Figure 1A).

The AV and POV were not significantly different between oil extracted from crackers and oil heated in the oven. However, the AV and POV of both oil extracted from crackers and oil heated in the oven were significantly higher than fresh oil, as shown in Figure 1B and Figure 1C. Moreover, the AV and POV were higher at 180 °C compared to 160 °C. Overall, the AV was below 0.8 mg/g and the POV did not exceed 0.3 g/100g in all oil samples.

In terms of fatty acid composition, there was no significant change in oleic acid (C18:1). Oleic acid, the main monounsaturated fatty acid found in olive oil, was increased by 0.14–0.23% after baking (Table 1). In addition, no harmful trans-fatty acids (TFA) were formed during baking.

### 3.2. Intermittent Frying of Chicken

Frying is a repetitive process and the heating duration is relatively longer than baking. The physico-chemical characteristics of the olive-based DAG oil were measured after 3 days of frying at 160 and 180 °C.

**Figure 2:**
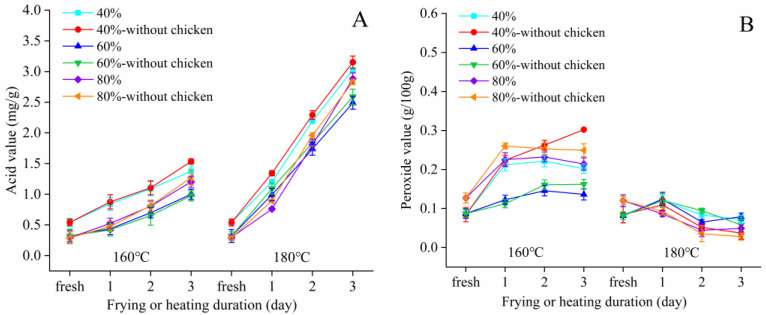
AV (**A**) and POV (**B**) of the 40, 60 and 80% olive-based DAG oil after 3 days of frying.

As expected, the AV increased proportionally as temperature increased, until it reached 3.30 mg/g (Figure 2A). With the extension of frying duration, the POV increased slowly and then decreased. Interestingly, the POV reached its peak value (0.23 g/100 g) on the first day and then decreased to 0.19 g/100 g (Figure 2B). In addition, there was no significant difference (*p* > 0.05) between frying chicken and control (without chicken).

During frying, the fatty acid composition changed significantly (Table 2). With the continuous heating, palmitic acid (C16:0) increased, and the content of oleic acid (C18:1), linoleic acid (C18:2) and linolenic acid (C18:3) decreased. Interestingly, C18:1t was detected after frying at 180 °C for 2 days, mainly because C18:1 was the most abundant unsaturated acid in the oils. In addition, C8:0 was detected on the third day at 160 °C and the second day at 180 °C.

### 3.3. The Effects of Salt, Duration, Temperature and DAG Content on 3-MCPDE and GE Content in Baking and Frying

When baking crackers with vegetable oil, the addition of salt affected the production of 3-MCPDE [13]. It can be observed in Table 3 that the content of 3-MCPDE was relatively low in oil heated in the oven (without salt and other materials) compared to the oil extracted from crackers. In other words, 3-MCPDE was 1.08 mg/kg for fresh oil, 0.97 mg/kg for oil extracted from crackers and 0.84 mg/kg for oil heated in the oven (40% DAG at 160 °C).

Table 4 clearly shows that 3-MCPDE decreased to 0.30 mg/kg, while the content of GE slightly increased with the prolonging of heating duration. The maximum content of GE was still below 1 mg/kg. However, a higher level of GE was found in the control after 3 days of frying (1.29 mg/kg). Similarly, under the condition of intermittent deep-frying, 3-MCPDE showed a significant decline, while GE showed an increasing trend of [9]. It was found that the formation of 3-MCPDE during long-term heating was lower than the rate of its decomposition, while GE increased and was relatively stable [7].

There was no correlation between the production of the contaminants and DAG content. The GE was below the limit of quantification with the increase in DAG content at 160 °C, even after long-term heating (Table 4). After 3 days of heating, the 3-MCPDE was the lowest in the 40% olive-based DAG oil (1.07 mg/kg).

### 3.4. Effect of Temperature, Duration and DAG Content on 3-MCPDE and GE

The influence of temperature, heating duration and DAG content on 3-MCPDE and GE were analyzed using the Pearson correlation test and PLS during baking and frying.

During baking, temperature showed a very strong correlation with 3-MCPDE (r > 0.9) and GE (r > 0.9), while the correlations were not significant between DAG content and both 3-MCPDE (P = 0.365) and GE (*p* = 0.563). In frying oil, temperature displayed a negative and very strong correlation with 3-MCPDE (r > 0.9) and a strong correlation with GE (0.7 < r < 0.9). Moreover, changes in 3-MCPDE and GE were correlated with the frying duration (r > 0.9). Interestingly, DAG showed moderate correlation with 3-MCPDE (0.5 < r < 0.7). On the other hand, the correlations were not significant between DAG content and GE (*p* = 0.754).

**Figure 3:**
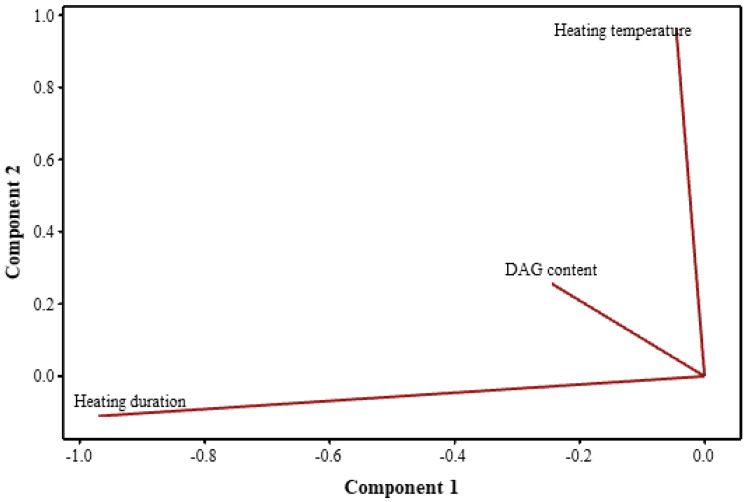
The loadings plot of the PLS model.

The PLS model employed had two components with an R^2^ value of 0.7775 and a p-value less than 0.05, thus authenticating its validity. The load diagram compares the relative influence of each prediction variable on the response. In this study, the line corresponding to DAG content was short, indicating the weak correlation with 3-MCPDE and GE (Figure 3). The lines of heating temperature and duration were very long, indicating that they were more relevant to 3-MCPDE and GE.

## 4. Discussion

The crackers were relatively lower in hardness and gumminess when baked at 160 °C, and there was no significant difference (*p* > 0.05) among the three groups of crackers. This might be caused by the emulsifying properties of DAG [16]. The physico-chemical characteristics in the baking trial were not significantly different (*p* > 0.05) between oil extracted from crackers and oil heated. This observation was related to the good thermal stability of high-oleic-acid oil at 180 °C [18].

During frying, the increase in AV can be explained by the increase in free fatty acids due to the hydrolysis reaction [25]. The reduced POV was caused by the decomposition and transformation of the primary oxidation product [26,27]. During frying, the fatty acid composition changed significantly (Table 2). There is a higher tendency of unsaturated oils to oxidation and polymerization reactions during frying or heating compared to oils with less unsaturation such as olive oil, which has more oleic fatty acid [28]. Based on previously reported data, heating could result in the isomerization of unsaturated fatty acids’ double bonds from cis to trans forms [29].

Salt, as a chlorine source, can provide a precursor for the formation of 3-MCPDE [30]. The results indicated that 3-MCPDE was generally lower in oil extracted from crackers and oil heated in the oven than in the fresh oil. In addition, the content of 3-MCPDE was higher in the presence of salt (oil extracted from crackers) under the same baking conditions. In the case of GE, the maximum value of GE was still below the limit of quantification (0.60 mg/kg) among the three groups, indicating that the presence of salt might not be related to the formation of GE in this study [11]. From the findings, 3-MCPDE decreased with the increase in temperature, while GE was lower at 160 °C (Table 4). During heating, GE increased to 1.29 mg/kg with the increase in temperature. Similarly, during deep-frying, 3-MCPDE declined, while GE showed an increasing trend [31]. Researchers have opined that the effect of heating duration is more relevant than temperature [4]. Merkle et al. found that there was no significant difference in the contaminants when frying fish with a mixture of MAG/DAG [32]. In addition, the production of 3-MCPD and free glycidol was confirmed in heated MAG, but not from DAG [12]. DAG could be a precursor in the heating process depending on the conditions.

The results of Pearson and PLS analyses suggest that DAG led to the formation of 3-MCPDE and GE in heat-induced processing and was closely related to the conditions, mainly heating temperature and duration. The order of the effects was duration > temperature > DAG content. The previous studies also found that the role of DAG in the formation of 3-MCPDE and GE was not clear, but high temperature was indeed the key factor [33].

## 5. Conclusions

It was concluded that olive-based DAG oil is safe and stable to be used in both baking and frying. The temperature and duration of heating treatment affect the physico-chemical properties of the olive-based DAG oil. As the temperature and duration increased, the AV continued to increase, the primary oxidation products were produced and rapidly transformed, and the decomposition of fatty acids was more likely to occur. The 3-MCPDE and GE in crackers and frying oil were lower than the EU limit (1.25 mg/kg and 1 mg/kg, respectively). Under the heating conditions of 160 and 180 °C, 3-MCPDE and GE showed a correlation with duration (r > 0.9). However, the content of DAG had no significant effect on 3-MCPDE and GE (r < 0.5). In conclusion, the factors in forming 3-MCPDE and GE were mostly associated with heating duration, followed by temperature and lastly DAG content.

## Figures and Tables

**Table 1:** Fatty acid composition of oil extracted from crackers and oil heated in the oven at 160 and 180 °C.

FA (%)	Fresh	Oil Extracted from Crackers	Oil Heated in Oven	Fresh	Oil Extracted from Crackers	Oil Heated in Oven
	40%–160 °C			40%–180 °C		
C16:0	10.55 ± 0.07a	10.63 ± 0.05a	10.68 ± 0.06a	10.55 ± 0.04a	11.50 ± 0.05a	11.45 ± 0.07a
C18:0	3.12 ± 0.04a	2.40 ± 0.05b	2.48 ± 0.05b	3.21 ± 0.01b	3.46 ± 0.02a	3.49 ± 0.01a
C18:1	72.61 ± 0.08a	72.78 ± 0.07a	72.71 ± 0.09a	72.11 ± 0.07a	72.23 ± 0.07a	72.07 ± 0.08a
C18:1t	ND	ND	ND	ND	ND	ND
C18:2	9.57 ± 0.05a	9.44 ± 0.07a	9.49 ± 0.05a	9.97 ± 0.04a	8.60 ± 0.05b	8.72 ± 0.02b
C18:3	0.94 ± 0.03a	1.02 ± 0.01a	1.01 ± 0.00a	0.94 ± 0.00b	1.27 ± 0.00a	1.30 ± 0.02a
	60%–160 °C			60%–180 °C		
C16:0	11.53 ± 0.05b	12.05 ± 0.05a	12.07 ± 0.03a	11.12 ± 0.01a	11.19 ± 0.05a	11.14 ± 0.03a
C18:0	3.15 ± 0.03a	3.08 ± 0.03a	3.11 ± 0.02a	3.13 ± 0.00b	3.31 ± 0.01a	3.36 ± 0.01a
C18:1	68.88 ± 0.09a	68.93 ± 0.08a	69.00 ± 0.05a	71.62 ± 0.05a	71.67 ± 0.08a	71.66 ± 0.07a
C18:1t	ND	ND	ND	ND	ND	ND
C18:2	11.72 ± 0.03a	11.45 ± 0.03b	11.38 ± 0.04b	11.14 ± 0.04a	10.29 ± 0.06b	10.19 ± 0.04b
C18:3	1.22 ± 0.03b	1.33 ± 0.02a	1.30 ± 0.01a	1.11 ± 0.01a	1.12 ± 0.00a	1.14 ± 0.01a
	80%–160 °C			80%–180 °C		
C16:0	11.18 ± 0.02a	11.12 ± 0.05a	11.12 ± 0.02a	11.14 ± 0.02b	11.94 ± 0.05a	12.00 ± 0.05a
C18:0	3.08 ± 0.03a	3.13 ± 0.02a	3.12 ± 0.01a	3.06 ± 0.02a	3.11 ± 0.03a	3.16 ± 0.02a
C18:1	69.47 ± 0.05a	69.42 ± 0.07a	69.51 ± 0.02a	68.89 ± 0.05a	68.91 ± 0.06a	69.00 ± 0.04a
C18:1t	ND	ND	ND	ND	ND	ND
C18:2	11.67 ± 0.05b	11.72 ± 0.02a	11.81 ± 0.05a	11.68 ± 0.03a	11.68 ± 0.01a	11.69 ± 0.05a
C18:3	1.12 ± 0.01a	1.10 ± 0.00a	0.99 ± 0.02a	1.25 ± 0.01a	1.22 ± 0.02a	1.21 ± 0.01a

Each value is the mean ± standard deviation of triplicate determinations. FA, Fatty acid composition. C16:0, palmitic acid. C18:0, stearic acid. C18:1, oleic acid. C18:1t, trans-oleic acid. C18:2, linoleic acid. C18:3, linolenic acid. ND, not detected. Different lowercase letters denote significant differences (*p* < 0.05) among fresh oil, oil extracted from crackers and oil heated in the oven.

**Table 2:** Changes in fatty acid composition of the 40, 60 and 80% olive-based DAG oil after frying for 3 days.

FA (%)	Day 1	Day 2	Day 3	Day 1	Day 2	Day 3
	40%–160 °C			40%–180 °C		
C8:0	ND	ND	0.47 ± 0.01	ND	0.43 ± 0.01	0.88 ± 0.01
C16:0	10.7 ± 0.10c	11.65 ± 0.14b	14.09 ± 0.11a	11.64 ± 0.09c	13.6 ± 0.10b	15.96 ± 0.07a
C18:0	2.41 ± 0.02b	2.67 ± 0.02b	3.81 ± 0.02a	3.54 ± 0.03c	4.09 ± 0.02b	5.11 ± 0.02a
C18:1	74.69 ± 0.42a	74.19 ± 0.35a	69.24 ± 0.44b	73.72 ± 0.42a	71.89 ± 0.40a	65.33 ± 0.58b
C18:1t	ND	ND	ND	ND	0.06 ± 0.01	0.24 ± 0.01
C18:2	8.96 ± 0.01a	6.63 ± 0.00b	2.49 ± 0.01c	7.57 ± 0.01a	3.64 ± 0.01b	1.18 ± 0.01c
C18:3	0.70 ± 0.00a	0.40 ± 0.00b	0.06 ± 0.00c	0.54 ± 0.00a	0.13 ± 0.00b	0.04 ± 0.00b
	60%–160 °C			60%–180 °C		
C8:0	ND	ND	0.37 ± 0.01	ND	0.42 ± 0.01	1.09 ± 0.01
C16:0	12.11 ± 0.12b	12.82 ± 0.15b	14.71 ± 0.13a	12.01 ± 0.09c	14.09 ± 0.10b	18.35 ± 0.09a
C18:0	3.78 ± 0.01b	3.26 ± 0.02b	4.01 ± 0.01a	3.34 ± 0.02b	3.92 ± 0.03b	5.23 ± 0.02a
C18:1	69.33 ± 0.55b	70.15 ± 0.35a	69.99 ± 0.43a	71.86 ± 0.44a	71.03 ± 0.42a	59.44 ± 0.59b
C18:1t	ND	ND	ND	ND	0.05 ± 0.01	0.23 ± 0.01
C18:2	12.11 ± 0.11a	10.13 ± 0.01b	5.65 ± 0.00c	11.06 ± 0.09a	4.50 ± 0.05b	0.71 ± 0.01c
C18:3	1.05 ± 0.00a	0.73 ± 0.00b	0.24 ± 0.00c	0.69 ± 0.01a	0.17 ± 0.00b	0.17 ± 0.00b
	80%–160 °C			80%–180 °C		
C8:0	ND	ND	0.38 ± 0.01	ND	0.36 ± 0.01	0.87 ± 0.01
C16:0	12.12 ± 0.12c	13.21 ± 0.15b	14.79 ± 0.10a	12.29 ± 0.09c	13.97 ± 0.11b	16.87 ± 0.09a
C18:0	3.13 ± 0.01b	3.46 ± 0.03b	4.20 ± 0.02a	3.26 ± 0.03c	3.70 ± 0.03b	4.52 ± 0.02a
C18:1	69.42 ± 0.41a	70.23 ± 0.37a	66.41 ± 0.54b	69.99 ± 0.49a	70.08 ± 0.48a	63.67 ± 0.50b
C18:1t	ND	ND	ND	ND	0.04 ± 0.00	0.09 ± 0.01
C18:2	11.72 ± 0.10a	8.79 ± 0.10b	5.25 ± 0.01c	10.61 ± 0.13a	6.54 ± 0.10b	2.25 ± 0.01c
C18:3	1.10 ± 0.00a	0.60 ± 0.00b	0.23 ± 0.00c	0.86 ± 0.00a	0.33 ± 0.00b	0.04 ± 0.00c

Each value is the mean ± standard deviation of triplicate determinations. FA, Fatty acid compositions. ND, not detected. C8:0, caprylic acid. C16:0, palmitic acid. C18:0, stearic acid. C18:1, oleic acid. C18:1t, trans-oleic acid. C18:2, linoleic acid. C18:3, linolenic acid. Different lowercase letters denote significant differences (*p* < 0.05) among heating duration.

**Table 3:** The 3-MCPDE and GE of oil extracted from crackers and oil heated in the oven.

	3-MCPDE (mg/kg)			GE (mg/kg)		
160 °C	Fresh	oil extracted from crackers	oil heated in the oven	Fresh	oil extracted from crackers	oil heated in the oven
40%	1.08 ± 0.01a	0.97 ± 0.02b	0.84 ± 0.00c	ND	ND	ND
60%	1.23 ± 0.02a	1.21 ± 0.02a	1.03 ± 0.07b	ND	ND	ND
80%	1.12 ± 0.07a	0.92 ± 0.05ab	1.01 ± 0.03a	ND	ND	ND
180 °C	Fresh	oil extracted from crackers	oil heated in the oven	Fresh	oil extracted from crackers	oil heated in the oven
40%	1.08 ± 0.01a	0.62 ± 0.01b	0.33 ± 0.01c	ND	ND	ND
60%	1.23 ± 0.02a	1.15 ± 0.05a	1.04 ± 0.02b	ND	<LOQ	ND
80%	1.12 ± 0.07a	0.69 ± 0.03b	0.63 ± 0.01b	ND	<LOQ	<LOQ

One-way analysis of variance was used to indicate significant differences between each oil sample (*p* < 0.05) as shown by different lowercase letters. LOQ, limit of quantification. ND, not detected (below the limit of quantification: GE LOQ = 0.60 mg/kg, GE LOD = 0.24 mg/kg).

**Table 4:** The 3-MCPDE and GE of oil after frying for 3 days.

Content (mg/kg)	40%-DAG		60%-DAG		80%-DAG	
	3-MCPDE	GE	3-MCPDE	GE	3-MCPDE	GE
Fresh	1.07 ± 0.01a	ND	1.23 ± 0.02a	ND	1.21 ± 0.01b	ND
160 °C						
Day 1	0.67 ± 0.02c	ND	1.20 ± 0.05a	ND	1.16 ± 0.01c	ND
Day 2	<LOQ	ND	1.09 ± 0.01b	ND	0.85 ± 0.02b	ND
Day 3	<LOQ	ND	1.07 ± 0.04b	ND	<LOQ	<LOQ
160 °C-without chicken						
Day 1	0.79 ± 0.02b	ND	1.12 ± 0.05a	ND	1.25 ± 0.01b	ND
Day 2	<LOQ	ND	1.07 ± 0.04b	ND	1.11 ± 0.02c	<LOQ
Day 3	<LOQ	<LOQ	0.81 ± 0.04c	<LOQ	0.92± 0.01e	<LOQ
180 °C						
Day 1	0.71 ± 0.01b	ND	0.99 ± 0.03b	ND	1.09 ± 0.01d	ND
Day 2	0.32 ± 0.06e	<LOQ	0.73 ± 0.01d	ND	0.91 ± 0.02e	<LOQ
Day 3	<LOQ	0.73 ± 0.02b	<LOQ	<LOQ	0.86 ± 0.03f	0.85 ± 0.02b
180 °C-without chicken						
Day 1	0.72 ± 0.01b	ND	0.98 ± 0.01b	ND	1.31± 0.02a	<LOQ
Day 2	0.46 ± 0.06d	<LOQ	0.51 ± 0.01e	<LOQ	1.15 ± 0.03c	0.73 ± 0.02c
Day 3	<LOQ	0.97 ± 0.02a	<LOQ	0.91 ± 0.03a	0.87± 0.04f	1.29 ± 0.05a

One-way analysis of variance was used to indicate significant differences between each frying interval (*p* < 0.05) as shown by different lowercase letters. LOQ, limit of quantification. ND, not detected. 3-MCPED LOQ = 0.30 mg/kg, 3-MCPED LOD = 0.10 mg/kg, GE LOQ = 0.60 mg/kg, GE LOD = 0.24 mg/kg.

## Data Availability

The data presented in this study are available on request from the corresponding author.
